# Left lower lobectomy without pericardial reconstruction in a patient with a congenital pericardium defect

**DOI:** 10.1093/icvts/ivad113

**Published:** 2023-07-08

**Authors:** Jun Miura, Hiroyuki Ito, Hiroyuki Adachi, Tetsuya Isaka

**Affiliations:** Department of Thoracic Surgery, Kanagawa Cancer Center, 2-3-2 Nakao, Asahi, Yokohama, Kanagawa, 241-8515, Japan; Department of Thoracic Surgery, Kanagawa Cancer Center, 2-3-2 Nakao, Asahi, Yokohama, Kanagawa, 241-8515, Japan; Department of Thoracic Surgery, Kanagawa Cancer Center, 2-3-2 Nakao, Asahi, Yokohama, Kanagawa, 241-8515, Japan; Department of Thoracic Surgery, Kanagawa Cancer Center, 2-3-2 Nakao, Asahi, Yokohama, Kanagawa, 241-8515, Japan

**Keywords:** Pericardial defect, Severe adhesion, Lung cancer, Lobectomy

## Abstract

Pericardial defects are rare congenital disorders. We report a case of a left lower lobectomy in a patient with lung cancer, a congenital complete left-sided pericardial defect and severe pleural adhesions. The pleural adhesions between the epicardium and lungs were carefully dissected. A left lower lobectomy with mediastinal nodal dissection was performed under complete video-assisted thoracoscopic surgery without pericardial reconstruction. The patient remained asymptomatic for 20 months postoperatively. Careful dissection of severe adhesions is necessary in patients with severe cardiac pulsations.

## INTRODUCTION

Complete pericardial defects are rare, asymptomatic congenital diseases incidentally detected during thoracic surgery [1]. The necessity of pericardial reconstruction during thoracic surgery in patients with complete pericardial defects remains controversial. We report a rare case of a complete congenital left-sided pericardial defect with severe pleural adhesions, detected during a left lower lobectomy, under complete video-assisted thoracoscopic surgery (cVATS) for lung cancer.

## CASE REPORT

**Figure 1:**
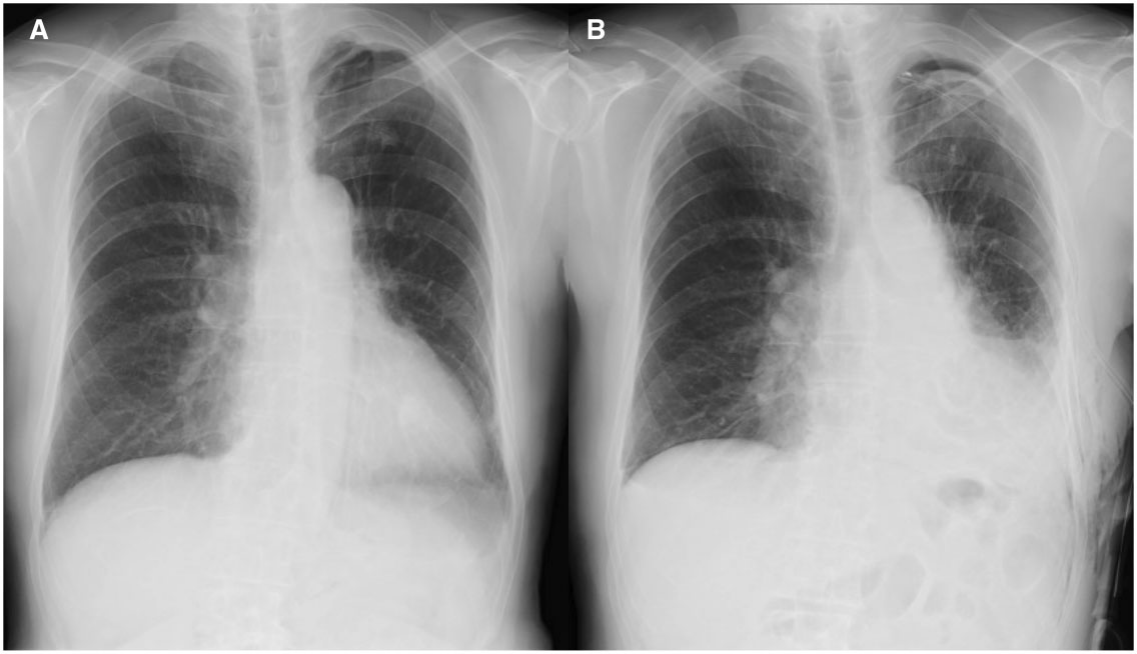
Pre- and postoperative chest radiographs. (**A**) The preoperative chest radiograph showed a leftward shift of the heart and a dull cardiac diaphragm. The trachea remained in the midline. (**B**) The postoperative chest radiograph showed no cardiac or mediastinal deviation.

A 75-year-old asymptomatic Japanese man was suspected of having cancer of the left lower lung lobe. The patient had a history of pericarditis and pneumonia. Chest radiography revealed a leftward shift of the heart and a dull cardiac diaphragm (Fig. 1A). Echocardiography revealed no abnormal wall motions.

**Figure 2:**
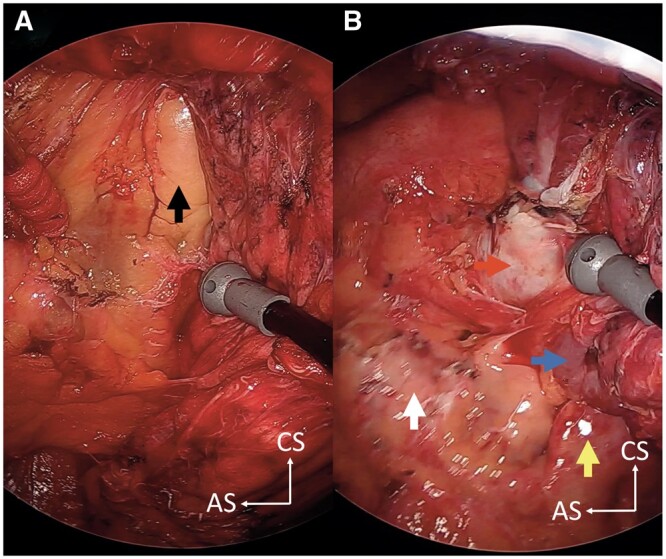
Surgical findings. (**A**) The pericardial adipose tissue (black arrow) was seen while we were dissecting the adhesions on the mediastinal side. (**B**) The left pericardium was completely deficient, and the left atrial auricle (yellow arrow) was exposed. Severe cardiac pulsations (white arrow) were observed. The left main pulmonary artery (red arrow) and left superior pulmonary vein (blue arrow) were also located. AS anterior side; CS cranial side.

Severe adhesions were observed, and total dissection of the pleural cavity was performed using cVATS. During the dissection of the anterior mediastinal side of the adhesion, distinguishing the epicardium and epicardial adipose tissue from the pericardial adipose tissue was initially difficult (Fig. 2A). Intense cardiac pulsations were observed, whereas the left pericardium was completely deficient. The patient was diagnosed with a complete left-sided pericardial defect (Fig. 2B). A lower left lobectomy with mediastinal lymph node dissection was performed using 5-port cVATS. The left phrenic nerve was detected on the ventral side of the mediastinum, behind the sternum. An air leak was alleviated via suture repair, and the injured lung parenchyma was reinforced with a polyglycolic acid sheet. The remaining segment of the upper lung lobe was fully expanded, and pericardial reconstruction was not performed. The operating time was 296 min, and blood loss was 100 ml. The continuous suction pressure of the drain was set to -5 cm H_2_O, using the Thopaz Digital Chest Drainage and Monitoring System (Medela, Baar, Switzerland), to avoid cardiac deviation due to postoperative intrapleural hypernegative pressure. Postoperative radiographs showed no mediastinal deviation (Fig. 1B). The patient was discharged without complications on postoperative day 6. He remained asymptomatic without signs of recurrence 20 months postoperatively.

## DISCUSSION

Pericardial defects occur in 1 out of 14,000 individuals. These defects result from pericardial dysplasia due to premature atrophy of Cuvier's canal [1].

Several studies have reported the development of severe adhesions during thoracic surgery for complete pericardial defects. Epicardial injury during the dissection of pleural adhesions causes fatal intraoperative cardiovascular events due to coronary vascular injury. The epicardial adipose tissue was similar to the pericardial adipose tissue. Therefore, myocardial injuries may occur when thoracic surgeons are unaware of complete pericardial defects. Careful dissection is necessary for patients with severe cardiac pulsation.

Pericardial reconstruction of the defects detected during thoracic surgery remains controversial. Yamaguchi *et al.* performed a pericardial reconstruction with (polypropylene) mesh during a left upper lobectomy. However, the reconstruction of the pericardium was repeated because of postoperative cardiac deviations [2]. In other studies, total left pneumonectomy [3] or left upper lobectomy [4] was performed without pericardial reconstruction in patients with complete left-sided pericardial defects. Reconstruction was reportedly unnecessary if the residual lower lung lobe expanded adequately during the upper lobectomy. Meanwhile, reconstruction should be considered after a lower lobectomy or pneumonectomy [4].

In the present case, pericardial reconstruction was not performed because the expansion of the residual left upper lobe was sufficient. Additionally, drug-induced adhesion procedures performed in the event of a prolonged air leak may have a negative impact on the myocardium. Therefore, suturing of the air leak site was performed carefully. The postoperative drainage pressure was decreased to avoid complications from cardiac ischaemic changes caused by postoperative cardiac deviations. The presence of adhesions in the contralateral pericardium does not always necessitate pericardial reconstruction because it limits cardiac deviation.

In conclusion, this report emphasizes the significance of performing a careful dissection of mediastinal adhesions in patients with intense cardiac pulsations.

## Data Availability

The data underlying this article are available in the article.
